# EZH2 Regulates the Pluripotency of Mouse Embryonic Stem Cells by Modulating *Nanog* Expression Under PKC Inhibition

**DOI:** 10.3390/biology15110880

**Published:** 2026-06-02

**Authors:** Fangfang Wu, Zhihui Liu, Yuan Gao, Jinshan Li, Xiao Chen, Xiyue Wang, Lanjun Liu, Fuliang Du

**Affiliations:** 1 School of Nursing and Health, Shanghai Zhongqiao Vocational and Technical University, Shanghai 200540, China; fangfangw@shzq.edu.cn; 2 College of Animal Science and Technology, Hebei Agricultural University, Baoding 071000, China; zhihuiliu@hebau.edu.cn; 3 Jiangsu Key Laboratory for Molecular and Medical Biotechnology, College of Life Sciences, Nanjing Normal University, Nanjing 210046, China; 221201021@njnu.edu.cn (Y.G.); lijinshan@163.com (J.L.); 231201021@njnu.edu.cn (X.C.); 4 Children’s Research Institute, University of Texas Southwestern Medical Center, Dallas, TX 75390, USA; xiyue.wang@utsouthwestern.edu; 5 Chengdu Institute of Biological Products Co., Ltd., 379, 3rd Section, Jinhua Road, Jinjiang District, Chengdu 610023, China

**Keywords:** embryonic stem cells, EZH2, *Nanog*, epigenetics

## Abstract

Polycomb repressive complex 2 (PRC2) regulates the expression of pluripotency genes in embryonic stem cells (ESCs) and suppresses multiple genes associated with development, cell fate determination, and differentiation. Mouse embryonic stem cells (mESCs) derived from protein kinase C inhibition (PKCi) exhibit self-renewal and pluripotency comparable to those ESCs captured by the classical 2iL (CHIR99021, PD0325901, and leukemia inhibitory factor) system. However, the dynamic expression pattern of PRC2 in PKCi-mESCs and its role in regulating pluripotency remain unclear. This study demonstrated that the expression level of the enhancer of zeste 2 gene (*Ezh2*), of which protein is the catalytic subunit of PRC2 responsible for the trimethylation of lysine 27 on nucleosome histone H3 subunit (H3K27me3), is significantly higher in PKCi-mESCs than in 2iL-mESCs. EZH2 knockdown enhances the self-renewal capacity of PKCi-mESCs, as evidenced by a significant increase in the number of undifferentiated mESCs colonies. The effect of an EZH2 reduced expression is accompanied by the upregulation of specific core pluripotency gene *Nanog*, along with the general downregulation of differentiational genes representing the three germ layers. Conversely, EZH2 overexpression promotes a significant differentiation of PKCi-mESCs, resulting in the downregulation of pluripotency genes, including core pluripotency genes *Nanog* and *Sox2*, as well as naïve pluripotency genes *Klf4*, *Fgf4*, and *Esrrb*, while with a wide upregulation of three germ layer associated genes. Importantly, Cleavage Under Targets and Tagmentation (CUT&Tag) demonstrates that EZH2 directly controls H3K27me3 enrichment at the *Nanog* promoter near the transcription start site. Thus, EZH2, a core subunit of PRC2, exhibits the distinct regulatory functions orchestrating mESCs at a poised state between self-renewal and differentiation under PKC inhibition. EZH2 exerts histone H3 methyltransferase activity to regulate *Nanog* expression as one of its key targets, thereby modulating the transcriptional regulatory network that maintains pluripotency and lineage specification in mESCs.

## Simple Summary

Protein kinase C inhibition (PKCi) supports the self-renewal and pluripotency of mouse embryonic stem cells (mESCs), but the epigenetic regulatory mechanism remains unclear. Polycomb repressive complex 2 (PRC2) and its catalytic subunit EZH2 mediate histone H3 lysine 27 trimethylation (H3K27me3) to govern gene expression. Here, we show that EZH2 expression is significantly higher in PKCi-mESCs than in 2iL-mESCs or mouse embryonic fibroblasts. The knockdown of EZH2 enhances self-renewal and upregulates the core pluripotency gene *Nanog* in PKCi-mESCs, while overexpression promotes differentiation and suppresses *Nanog*. CUT&Tag analysis reveals that EZH2 directly controls H3K27me3 enrichment at the *Nanog* promoter. Our results demonstrate that EZH2 modulates the balance between self-renewal and differentiation in PKCi-mESCs by epigenetically regulating *Nanog* expression, highlighting a context-dependent epigenetic mechanism that sustains pluripotency under PKC inhibition.

## 1. Introduction

Embryonic stem cells (ESCs) are pluripotent cells that are derived from the inner cell mass of mammalian blastocysts cultured in vitro [1]. Currently, ESCs with a germ line transmission capability can be obtained through the extracellular regulated protein kinases/glycogen synthase kinase-3 (ERK/GSK3) and leukemia inhibitory factor/signal transducer and activator of transcription (LIF/STAT3) signaling pathway, known as 2iL (CHIR99021, PD0325901, and LIF) [2,3], as well as the inhibition of protein kinase C (PKCi) [4,5]. Studies have revealed that treatment with the PKC inhibitor Gö6983 suppresses the PKCζ-NF-ĸB-miRNA-21/miRNA-29 signaling axis to maintain ESCs self-renewal [4]. Additionally, PKCi regulates the epigenetic modifications at the chromatin regions of stem cell-specific pluripotency genes, thereby sustaining naïve pluripotency gene expression [4].

It is well known that the 2iL system, as a classical model for studying naïve state mouse embryonic stem cells (mESCs), has been extensively investigated regarding its mechanisms in maintaining self-renewal and differentiation potential. In contrast, the PKCi system provides a novel strategy for the derivation and maintenance of mESCs with enhanced naïve pluripotency features. Although mESCs cultured under serum/LIF conditions also support germline transmission, PKCi-cultured mESCs display superior naïve state properties [5,6]. Specifically, these PKCi-derived mESCs demonstrate the upregulated expression of the naïve-state marker *Esrrb*, concurrent with the downregulation of primed-state markers *Fgf5* and *Dnmt3b* [6]. We have also reported that the regulatory nucleosome remodeling and deacetylation (NuRD) complex and its MBD3 subunit influence the naïve pluripotency of mESCs under PKCi [7].

Epigenetic factors influence gene expression by regulating cell-specific transcriptional states and via the recruitment to chromatin to establish a higher-order genomic topology and nucleosome density [8,9]. These mechanisms primarily modulate gene expression through DNA methylation, histone modifications, higher-order chromatin structure, and non-coding RNAs [10], enabling heritable changes in gene function without altering DNA sequences and resulting in phenotypic variation. Epigenetic regulation also serves as a key determinant of pluripotent stem cell differentiation and lineage specification [11]. Polycomb repressive complexes (PRCs), which function as gene-repressive regulators and are basically classified into Polycomb repressive complex 1 (PRC1) and Polycomb repressive complex 2 (PRC2), regulate gene expression during early development and contribute to the development of adult organisms [12]. PRCs also participate in stem cell identity and regulation [13,14]. PRC2 complexes, evolutionally conserved from *Drosophila* to mammals [15], primarily consists of the core subunits enhancer of zeste 1 (EZH1), enhancer of zeste 2 (EZH2), embryonic ectoderm development (EED), suppressor of zeste 12 (SUZ12), retinoblastoma-binding protein 4 (RBBP4), and retinoblastoma-binding protein 7 (RBBP7) [15]. EZH1 and EZH2 possess histone methyltransferase activity [16]. Through the catalytic subunits of EZH1 and/or EZH2, PRC2 mediates the methylation of lysine 27 on the histone H3 nucleosome subunit (H3K27me1, H3K27me2, and H3K27me3) modifications; it is involved in diverse biological processes, including the maintenance of cellular identity, proliferation, stem cell plasticity, and subsequent cell differentiation [13,16,17,18]. Compared with EZH1, EZH2 exhibits higher methyltransferase activity, and the H3K27me3 level in ESCs is primarily established through EZH2-mediated enzymatic activity [19]. EED recruits PRC1 complexes to H3K27me3-marked loci and enhances PRC1-mediated H2A ubiquitin E3 ligase activity [20]. Although EED lacks catalytic function, it serves as a scaffold protein that stabilizes the complex and physically connects EZH2 with histone H3 substrates [21,22,23]. SUZ12 is essential for PRC2 activity, whereas RBBP4/7 is not required for complex activity [24]. EZH2, EED, and SUZ12 constitute the minimal set of PRC2 subunits capable of executing histone methyltransferase activity and initiating gene repression [25,26]. In mESCs, EZH2 knockout impairs mesodermal differentiation; the dual knockout of EZH1 and EZH2 substantially reduces H3K27me3 deposition at Polycomb target sites [27]. EZH2 directs the neural fate specification by repressing competing mesodermal and endodermal programs, as indicated by the aberrant reactivation of mesodermal and endodermal genes during neural induction in EZH2-knockout human ESCs [18]. EZH2 also regulates early murine development, such that knockout embryos exhibit severe gastrulation defects. Finally, the loss of EZH2 prevents the establishment of ESCs [28].

A specific pluripotency gene regulatory network, comprising *Oct4*, *Nanog*, *Sox2*, *Esrrb*, *Klf4*, and others, characterizes ESCs in the ground state of pluripotency [29,30]. The regulation of stem cells between the state of pluripotency and differentiation involves reciprocal interactions between pluripotency-associated transcription factors and PRCs [31]. In ESCs, the pluripotent state is primarily governed by the core transcription factor triad octamer-binding transcription factor 4 (OCT4), SRY-box transcription factor 2 (SOX2), and *Nanog* homeobox (NANOG) [32]. Among these factors, NANOG is indispensable for establishing pluripotent ESCs [33] and can maintain mESC pluripotency in the absence of LIF [34,35]. Furthermore, NANOG promotes the proliferation of undifferentiated human ESCs and facilitates pluripotency induction [34]. In mESCs and induced pluripotent stem cells, EZH2 directly modulates the epigenetic state of the *Nanog* promoter and disrupts the balance of *Nanog* expression, thereby influencing the equilibrium between self-renewal and differentiation [36]. Although PRC2 plays a critical role in maintaining the balance between self-renewal and differentiation in ESCs, its mechanistic involvement in regulating PKCi-derived mESCs self-renewal remains poorly understood. Here, we examined the expression patterns of PRC2 core subunits (EZH2, EZH1, SUZ12, EED, RBBP4, and RBBP7) in mESCs under PKC inhibition, revealing the effects of PKCi on the expression of PRC2 components. Subsequently, by knocking down or overexpressing the core catalytic subunit EZH2, we investigated how the catalytic component of PRC2 complexes, fine-tuned, orchestrates self-renewal and pluripotency in mESCs under PKC inhibition.

## 2. Materials and Methods

Chemicals and reagents

Unless otherwise stated, all chemicals and reagents were purchased from Sigma-Aldrich (St. Louis, MO, USA).

### 2.1. Animal Maintenance, Superovulation, and Embryo Collection

C57BL/6J female mice aged 6–8 weeks were used for all experiments. All animal procedures were approved by the Animal Care and Use Committee of Nanjing Normal University (IACUC-20201209) and conducted in accordance with the guidelines of the United States National Institutes of Health. Female C57BL/6J mice received an intraperitoneal injection of 7.5 IU pregnant mare serum gonadotropin (Ningbo Second Hormone Factory, Cixi, China). After 48 h, 7.5 IU of human chorionic gonadotropin (Ningbo Second Hormone Factory) were administered. Female mice were then housed with healthy male mice at a 1:1 ratio, and the presence of a vaginal plug was confirmed the following morning. Mice were euthanized by cervical dislocation on day 3.5 after detection of the vaginal plug. Uteri were carefully collected and flushed with M2 culture medium to isolate blastocysts.

### 2.2. De Novo Derivation of mESCs

Blastocysts were seeded on 0.1% gelatin-coated culture dishes (ES-006-B, Millipore, Burlington, MA, USA) containing mitomycin C–pretreated mouse embryonic fibroblasts (MEF) isolated from C57BL/6J mouse embryos at embryonic day 12.5–13.5. Outgrowths were observed after 1 week of culture and subsequently passaged using Accutase (09720, STEMCELL Technologies, Vancouver, BC, Canada). Both the 2iL and PKCi culture systems were utilized for blastocyst and mESC culture under 5% CO_2_ at 37 °C. mESCs were cryopreserved in a solution composed of 90% fetal bovine serum and 10% dimethyl sulfoxide. The PKCi culture system was based on KNOCKOUT Dulbecco’s Modified Eagle Medium (DMEM) supplemented with 15% KNOCKOUT Serum Replacement (KOSR, 10828028, Gibco, Grand Island, NY, USA), 7.5 μM Gö6983 (33053-19-7, Selleck, Houston, TX, USA), 1% penicillin/streptomycin (SV30010, HyClone, Logan, UT, USA), 1 mM sodium pyruvate (11360088, Gibco), 0.1 mM β-mercaptoethanol (ES-007-E, Millipore), and 2 mM GlutaMax (35050-061, Gibco). The 2iL culture system used KNOCKOUT DMEM as the basal medium, supplemented with 15% KOSR, 1% penicillin/streptomycin, 1 mM sodium pyruvate, 0.1 mM β-mercaptoethanol, 2 mM GlutaMax, 10^3^ IU/mL murine LIF (ESG1107, Millipore, Burlington, MA, USA), 1 μM PD0325901 (S1036, Selleck, Houston, TX, USA), and 3 μM CHIR99021 (CT99021, Selleck).

### 2.3. RNA Extraction, cDNA Synthesis, and Quantitative Polymerase Chain Reaction (qPCR) Analysis

Total RNA was extracted using VeZol reagent (R411, Vazyme, Nanjing, China). cDNA synthesis was performed via reverse transcription using the ABScript III RT Master Mix for qPCR (RK20429, ABclonal, Wuhan, China). qPCR was carried out with the Genious 2X SYBR Green Fast qPCR Mix (RK21207, ABclonal), in accordance with the manufacturer’s instructions. Gene expression levels were normalized to the housekeeping gene *β-Actin*. All primers used are listed in Table 1.

### 2.4. Total Protein Preparation and Western Blot Analysis

Total protein for western blot analysis was extracted using radioimmunoprecipitation assay buffer (C500005, Sangon, Shanghai, China). Briefly, cell lysates were incubated on ice for 5–10 min. During this period, vigorous vortexing was performed 3 to 4 times, each lasting 30 s. Cell extracts were centrifuged at 13,400× *g* for 15 min at 4 °C. Protein concentrations were measured using the bicinchoninic acid assay (E112-01, Vazyme), following the manufacturer’s instructions. Protein samples were separated by sodium dodecyl sulfate–polyacrylamide gel electrophoresis using 10% or 12% Tris-HCl gels at 120 V. They were subsequently transferred onto polyvinylidene fluoride membranes (03010040001, Roche, Basel, Switzerland) at 300 mA for 80–120 min at 4 °C in transfer buffer. Membranes were blocked with 5% non-fat milk (A600669, Sangon) in Tris-buffered saline containing 0.1% Tween 20 (TBST) for 1 h at room temperature with gentle rotation. Blocked membranes were incubated overnight at 4 °C with the appropriate primary antibody under rotation. The following day, membranes were washed three times with TBST for 10 min each, then incubated with horseradish peroxidase–conjugated goat anti-rabbit secondary antibody (1:5000, BS13278, Bioworld Technology, Minneapolis, MN, USA) diluted in TBST for 2 h at room temperature. After three additional TBST washes at room temperature, protein bands were visualized using the SuperPico ECL Chemiluminescence Kit (E422-01, Vazyme). Details of primary and secondary antibodies are listed in Table 2.

### 2.5. Lentivirus Production and Cell Infection

Lentivirus was produced by transfecting 293T packaging cells (1.0–1.5 × 10^6^ cells per 60 mm dish) with 3 µg of psPAX, 2 µg of pMD2.G, and 5 µg of plasmid (shEZH2 interference plasmids or oe-EZH2 overexpression plasmids) using a liposome-based transfection method. The control group was transduced with non-targeting scrambled shRNA for EZH2 knockdown experiments and empty lentiviral vector for EZH2 overexpression experiments. Cells were incubated with the transfection reagent (11668019; Invitrogen, Carlsbad, CA, USA) for 6–8 h, after which the medium was replaced with fresh PKCi-mESCs medium. Lentiviral particles were collected at 48 h and 72 h post-transfection and then filtered through a 0.45 μm membrane filter (FF365-10pcs, Beyotime, Shanghai, China). For infection, mESCs at 70–80% confluence were cultured in PKCi medium supplemented with filtered lentiviral particles. After 24 h, the medium was replaced with fresh PKCi medium; cell samples were collected after an additional 48 h of culture. The target sequence for shEZH2 were GCACAAGTCATCCCGTTAAAG and GCAAATTCTCGGTGTCAAACA.

### 2.6. Alkaline Phosphatase (AP) Staining

AP staining was performed with the AP chromogenic kit (REF 11745832910, Roche, Basel, Switzerland), in accordance with the manufacturer’s protocol. Briefly, mESCs were fixed with 4% paraformaldehyde for 15 min at room temperature. Cells were stained with 1× 5-bromo-4-chloro-3-indolyl phosphate (BCIP)/nitro blue tetrazolium chromogenic solution for 30 min at room temperature in the dark, and then imaged under a microscope. Colonies of 50–100 μm in diameter were counted as single colonies, while those larger than 100 μm were quantified via volume ratio and converted into equivalent colony numbers. Based on AP staining, colonies with ≥90% AP-positive area were regarded as undifferentiated colonies, those with ≤20% as differentiated colonies, and those with 20–90% as mixed colonies.

### 2.7. Cleavage Under Targets and Tagmentation (CUT&Tag)

Chromatin samples were prepared from PKCi-mESCs under three conditions: untreated control, EZH2 knockdown, and EZH2 overexpression. Sequence libraries were generated using the Hyperactive Universal CUT&Tag Assay Kit (TD904, Vazyme), following the manufacturer’s instructions. DNA libraries were assessed for quality and quantified using the Agilent 2100 Bioanalyzer (Santa Clara, CA, USA), then utilized for cluster generation and sequencing on the Illumina NovaSeq 6000 platform (San Diego, CA, USA; read length: 150 bp). Adaptor sequences and low-quality bases (Phred score < 30) were removed using the Trim Galore software package (v0.6.10). The resulting FASTQ files were aligned to the mm39 reference genome using Bowtie2. Duplicate reads were removed with the Picard tool MarkDuplicates, and sequencing data were quantified and visualized using deepTools. For peak calling of ChIP-enriched regions relative to input controls, MACS2 software (v2.2.9.1) was used with the following parameters: macs2 callpeak -n Sample -g mm -B -p 0.05. Annotation, comparison, and visualization of the identified peaks were performed using ChIPseeker [37], considering the ±5 kb region around the peak center. Visualization of mapped reads in representative genomic regions was carried out using IGV software (v2.19) [38].

### 2.8. Data Access

The raw sequence data reported in this paper have been deposited in the Genome Sequence Archive in National Genomics Data Center, China National Center for Bioinformation/Beijing Institute of Genomics, Chinese Academy of Sciences (GSA: CRA026488), which are publicly accessible at https://ngdc.cncb.ac.cn/gsa/browse/CRA026488 (accessed on 28 May 2026).

### 2.9. Statistical Analysis

All experiments were performed using three independent PKCi-mESC clones as biological replicates per group to ensure results are generalizable and not attributable to clone-specific artifacts. Differences between two groups were assessed using independent sample *t*-tests. Comparisons among three groups were evaluated using one-way analysis of variance (ANOVA) followed by Tukey’s honestly significant difference (HSD) post hoc test for multiple comparisons. Data are presented as mean ± standard error of the mean (SEM). Statistical significance thresholds were regarded as *p* < 0.05 (*), *p* < 0.01 (**), and *p* < 0.001 (***).

## 3. Results

### 3.1. PKC Inhibition Promotes the Expression of PRC2 Core Subunits

**Figure 1:**
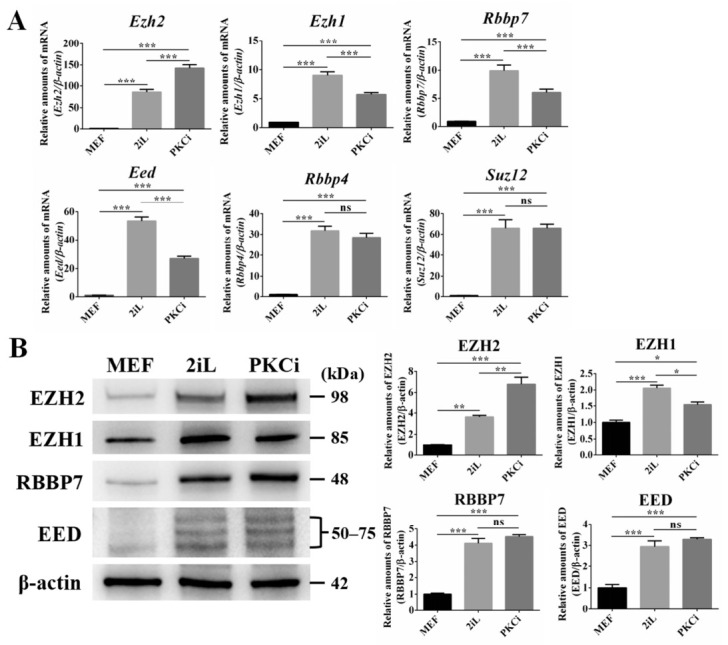
Gene expression patterns of PRC2 core components. (**A**) qPCR analysis showed that the mRNA expression levels of *Ezh1*, *Ezh2*, *Eed*, *Rbbp4*, *Rbbp7*, and *Suz12* in MEF, 2iL-mESCs, and PKCi-mESCs. (**B**) (See Supplementary Figure S1.) Representative Western blots showing protein levels of EZH2, EZH1, RBBP7, and EED in MEF, 2iL-mESCs, and PKCi-mESCs. MEF: fibroblast control; 2iL: mESC control. Data are presented as mean ± SEM (*n* = 3). Statistical significance thresholds were regarded as *p* < 0.05 (*), *p* < 0.01 (**), and *p* < 0.001 (***).

To examine the expression patterns of PRC2 core subunits in mESCs cultured under PKCi conditions, we performed a qPCR measurement of mRNA levels, focusing on the core subunit genes of PRC2, *Ezh1*, *Ezh2*, *Rbbp7*, *Rbbp4*, *Suz12*, and *Eed*. The expression levels of all six genes were significantly higher in 2iL-mESCs and PKCi-mESCs than in differentiated MEF (Figure 1A, *p* < 0.05). Notably, in the two mESC culture conditions, PKCi-mESCs exhibited a 66% increase in *Ezh2* mRNA levels compared with 2iL-mESCs (Figure 1A, *p* < 0.05), while displaying a reduced expression of *Ezh1* (37% lower), *Rbbp7* (39% lower), and *Eed* (50% lower) (Figure 1A, *p* < 0.05). No significant differences in *Suz12* or *Rbbp4* expression were observed between the two culture conditions. To further evaluate these findings at the protein level, a Western blot analysis was conducted for genes showing differential mRNA expression. The protein level of EZH2 was significantly higher (86% increase) in PKCi-mESCs than in 2iL-mESCs (Figure 1B, *p* < 0.05), consistent with the mRNA expression data. Conversely, the EZH1 protein level was significantly lower (25% decrease) in PKCi-mESCs (Figure 1B, *p* < 0.05). No significant differences in the protein levels of RBBP7 and EED were detected between the two mESC culture systems (Figure 1B).

### 3.2. EZH2 Knockdown Enhances Pluripotency Gene Expression and Suppresses Germ-Layer-Differentiation-Related Genes in PKCi-mESCs

**Figure 2:**
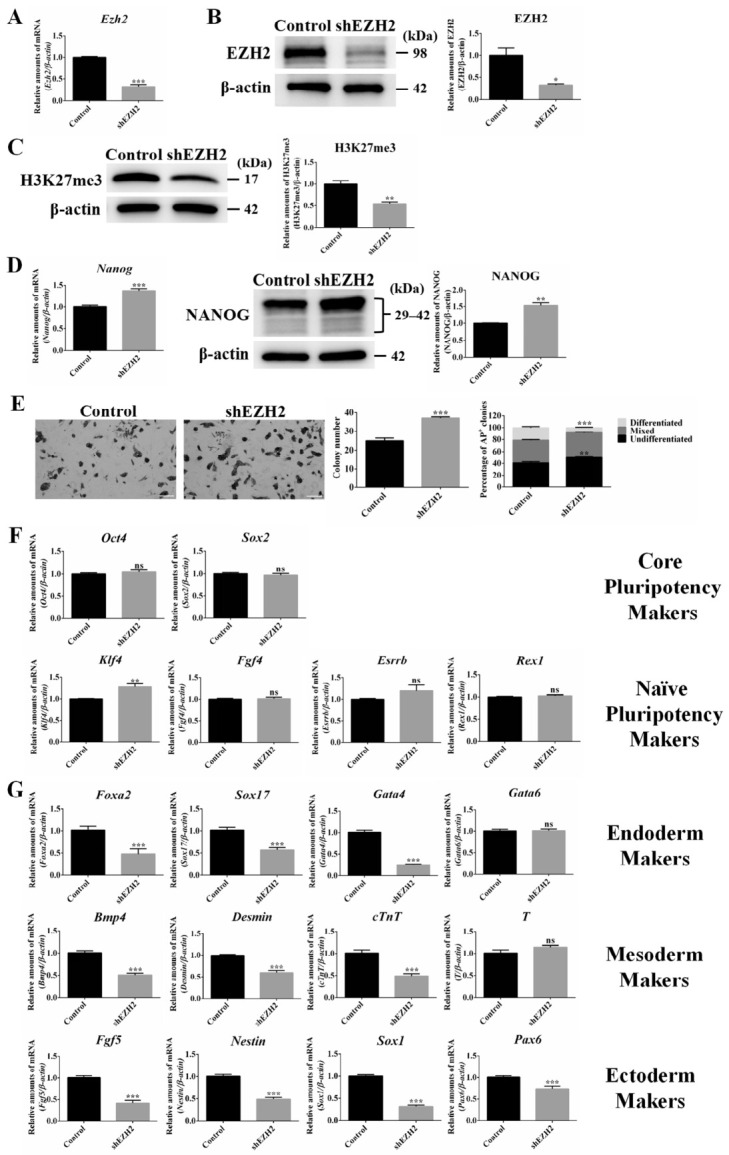
Effect of EZH2 knockdown on the expression of pluripotency genes and germ-layer-associated genes in PKCi-mESCs. (**A**) qPCR analysis of *Ezh2* mRNA expression in control and shEZH2 PKCi-mESCs. (**B**) (See Supplementary Figure S2.) Representative Western blots showing EZH2 protein levels after EZH2 knockdown. (**C**) (See Supplementary Figure S3.) Representative Western blots showing H3K27me3 modification levels after EZH2 knockdown. (**D**) qPCR analysis of *Nanog* mRNA expression after EZH2 knockdown. Representative Western blots showing NANOG protein expression after EZH2 knockdown (see Supplementary Figure S4). (**E**) AP staining of PKCi-mESCs. Scale bar: 200 μm. (**F**) qPCR analysis of mRNA expression of core pluripotency genes and naïve-state-specific marker genes. (**G**) qPCR analysis of germ-layer-associated expression. Control: PKCi-mESCs transduced with non-targeting scrambled shRNA; shEZH2: PKCi-mESCs with EZH2 knockdown. Data are presented as mean ± SEM (*n* = 3). Statistical significance thresholds were regarded as *p* < 0.05 (*), *p* < 0.01 (**), and *p* < 0.001 (***).

We found that PKC inhibition upregulated the expression of EZH2, the core catalytic subunit of PRC2. To explore the role of EZH2 in PKCi-mESCs, we knocked down EZH2 via lentiviral infection. Compared with the control group, EZH2 knockdown resulted in a 68% reduction in *Ezh2* mRNA levels (Figure 2A, *p* < 0.05) and a 67% decrease in EZH2 protein expression (Figure 2B, *p* < 0.05). EZH2 functions as a methyltransferase and primarily determines global H3K27me3 levels [19]. Our results demonstrated that EZH2 knockdown reduces H3K27me3 levels by 45% (Figure 2C, *p* < 0.05). In ESCs, NANOG maintains self-renewal by repressing the expression of developmental differentiation genes [33]. EZH2 mediates gene silencing through H3K27me3 modification [39] and directly regulates NANOG expression [36]. qPCR and Western blot analyses further confirmed that EZH2 knockdown resulted in a 37% upregulation in *Nanog* mRNA levels (Figure 2D, *p* < 0.05) and a 53% upregulation in NANOG protein expression (Figure 2D, *p* < 0.05). These findings indicate that EZH2 knockdown decreases H3K27me3 modification levels and promotes NANOG expression.

Additionally, AP staining revealed that EZH2 knockdown significantly increased the total number of AP-positive colonies among PKCi-mESCs (Figure 2E, *p* < 0.05), elevated the proportion of undifferentiated colonies from 40.9% to 50.2%, and reduced the proportion of differentiated colonies from 21.1% to 8.6% (Figure 2E, *p* < 0.05). These results suggest that EZH2 knockdown promotes an undifferentiated state in PKCi-mESCs. We hypothesized that the phenotypic changes might be related to the upregulation of pluripotency gene expression. To investigate the basis of this phenotypic change, we further analyzed the expression patterns of pluripotency genes and germ-layer-associated genes in EZH2-knockdown PKCi-mESCs via qPCR. EZH2 knockdown not only significantly increased the mRNA expression levels of the core pluripotency gene *Nanog* (37% higher) (Figure 2D, *p* < 0.05), but also increased the mRNA expression levels of naïve-state-specific marker gene *Klf4* (27% higher) (Figure 2F, *p* < 0.05). But no significant differences were observed in the expression of the core pluripotency gene *Oct4 and Sox2* or the naïve-state-specific marker gene *Fgf4*, *Esrrb,* and *Rex1* (Figure 2F). After EZH2 knockdown, the expression levels of multiple germ-layer-differentiation-related genes were significantly reduced (Figure 2G, *p* < 0.05). Specifically, among endodermal genes, *Foxa2* (53% decrease), *Sox17* (44% decrease), and *Gata4* (75% decrease) were downregulated. Among mesodermal genes, *Bmp4* (50% decrease), *Desmin* (40% decrease), and *cTnT* (52% decrease) were downregulated. Among ectodermal genes, *Fgf5* (59% decrease), *Nestin* (51% decrease), *Sox1* (68% decrease), and *Pax6* (26% decrease) were also significantly downregulated. Notably, no significant differences were observed regarding the expression patterns of the endodermal gene *Gata6* or the mesodermal genes *T* (Figure 2G). These results indicate that EZH2 modulates the self-renewal capacity of PKCi-mESCs in association with the altered expression of pluripotency genes and germ-layer-associated genes. Notably, although EZH2 knockdown decreased global H3K27me3 levels—a modification canonically linked to the derepression of the developmental genes—we observed the downregulated expression of multiple differentiation-associated markers. This suggests that, in PKCi-mESCs, the regulation of these genes is not solely dependent on H3K27me3 and may involve additional layers of control, including indirect effects via upregulated pluripotency factors such as NANOG.

### 3.3. EZH2 Overexpression Suppresses Pluripotency Gene Expression and Promotes Expression of Germ-Layer-Associated Genes in PKCi-mESCs

**Figure 3:**
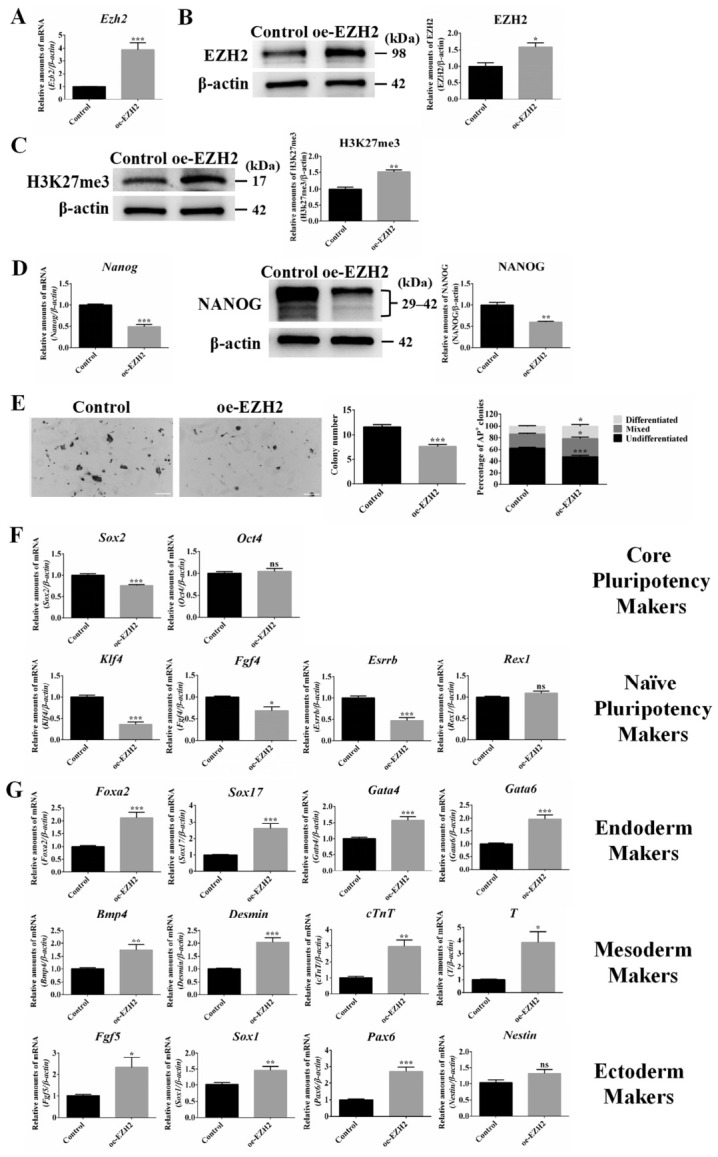
Effect of EZH2 overexpression on the expression levels of pluripotency genes and germ-layer-associated genes in PKCi-mESCs. (**A**) qPCR analysis of *Ezh2* mRNA expression in control and oe-EZH2 PKCi-mESCs. (**B**) (See Supplementary Figure S5.) Representative Western blots showing EZH2 protein levels after EZH2 overexpression. (**C**) (See Supplementary Figure S6.) Representative Western blots showing H3K27me3 modification levels after EZH2 overexpression. (**D**) qPCR analysis of *Nanog* mRNA expression after EZH2 overexpression. Representative Western blots showing NANOG protein expression after EZH2 overexpression (see Supplementary Figure S7). (**E**) AP staining of PKCi-mESCs. Scale bar: 200 μm. (**F**) qPCR analysis of core pluripotency genes (*Nanog*, and *Sox2*) and naïve-state-specific marker genes (*Klf4*, *Fgf4*, and *Esrrb*). (**G**) qPCR analysis of germ-layer-associated gene expression after EZH2 overexpression. Control: PKCi-mESCs transduced with empty lentiviral vector; oe-EZH2: PKCi-mESCs with EZH2 overexpression. Data are presented as mean ± SEM (*n* = 3). Statistical significance thresholds were regarded as *p* < 0.05 (*), *p* < 0.01 (**), *p* < 0.001 (***).

Given that EZH2 knockdown enhances the expression of core pluripotency genes and suppresses differentiation-related genes in PKCi-mESCs, we hypothesized that EZH2 overexpression negatively regulates self-renewal in this context. EZH2 overexpression via lentiviral infection increased *Ezh2* mRNA expression by 3.9-fold (Figure 3A, *p* < 0.05) and EZH2 protein levels by 1.6-fold (Figure 3B, *p* < 0.05) compared with the control group. Our data also show that EZH2 overexpression increases H3K27me3 levels by 1.5-fold (Figure 3C, *p* < 0.05), indicating that EZH2 overexpression promotes H3K27me3 accumulation. qPCR and Western blot analyses further confirmed that EZH2 overexpression resulted in a 51% reduction in *Nanog* mRNA levels (Figure 3D, *p* < 0.05) and a 40% decrease in NANOG protein expression (Figure 3D, *p* < 0.05). These findings suggest that EZH2 overexpression elevates H3K27me3 levels and suppresses NANOG expression. The downregulation of NANOG reduces its inhibitory effect on developmental differentiation genes, thereby facilitating ESC differentiation [33].

AP staining revealed that EZH2 overexpression significantly reduced the total number of AP-positive colonies (Figure 3E, *p* < 0.05). Specifically, the proportion of undifferentiated colonies decreased from 62.5% to 47.3%, whereas the proportions of mixed and differentiated colonies increased from 23.8% to 31.3% and 13.7% to 21.4%, respectively (Figure 3E, *p* < 0.05). These results indicate that EZH2 overexpression promotes differentiation in PKCi-mESCs. Subsequently, we analyzed the expression patterns of other pluripotency genes and germ-layer-associated genes after EZH2 overexpression in PKCi-mESCs. The results showed that the core pluripotency genes *Sox2* (24% decrease) and the naïve-state-specific marker genes *Klf4* (64% decrease), *Fgf4* (32% decrease), and *Esrrb* (53% decrease) were significantly downregulated (Figure 3F, *p* < 0.05). Furthermore, EZH2 overexpression significantly upregulated multiple genes involved in germ layer differentiation (Figure 3G, *p* < 0.05). Among endodermal genes, *Foxa2* (110% increase), *Sox17* (160% increase), *Gata6* (96% increase), and *Gata4* (57% increase) were upregulated. Among mesodermal genes, *Bmp4* (74% increase), *Desmin* (120% increase), *cTnT* (190% increase), and *T* (280% increase) showed significant upregulation. Regarding ectodermal genes, all except *Nestin* were significantly upregulated, including *Fgf5* (130% increase), *Sox1* (44% increase), and *Pax6* (170% increase) (Figure 3G, *p* < 0.05).

### 3.4. Epigenetic Regulation of Nanog Expression by H3K27me3

**Figure 4:**
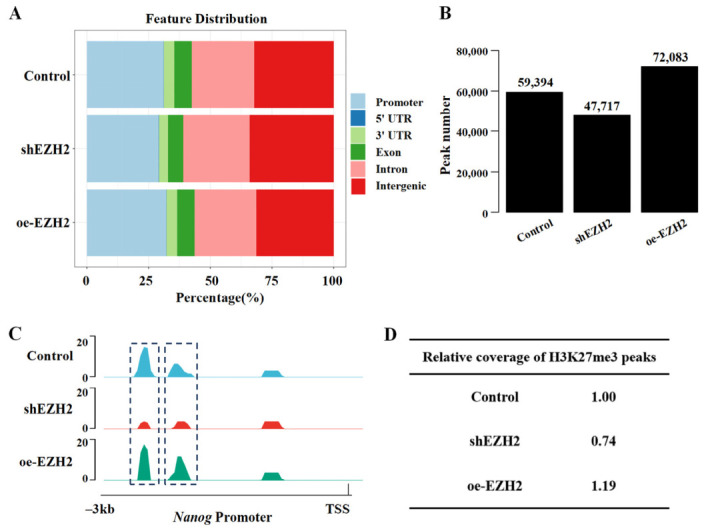
H3K27me3 CUT&Tag analysis of PKCi-mESCs treated with EZH2 knockdown or overexpression. (**A**) Distribution of H3K27me3 CUT&Tag peaks across key genomic regions in control, shEZH2, and oe-EZH2 PKCi-mESCs. (**B**) The number of H3K27me3 signal peaks among the sample of control, shEZH2, and oe-EZH2 PKCi-mESCs. (**C**) Genome browser view of H3K27me3 peaks at the *Nanog* promoter (−3 kb to 0 kb relative to the TSS). (**D**) Relative coverage of H3K27me3 peaks in *Nanog* promoter. The peak value of the control group was set as 1. Control: PKCi-mESCs; shEZH2: PKCi-mESCs with EZH2 knockdown; oe-EZH2: PKCi-mESCs with EZH2 overexpression. Data are presented as mean ± SEM (*n* = 3).

To determine whether the changes in *Nanog* expression after EZH2 perturbation are associated with the altered H3K27me3 deposition, we performed an H3K27me3 CUT&Tag analysis in control, EZH2-knockdown, and EZH2-overexpressing PKCi-mESCs. Genome-wide peak analysis showed that EZH2 perturbation was accompanied by changes in the number of detected H3K27me3 peaks. EZH2 knockdown reduced the number of H3K27me3 peaks from 59,394 in control cells to 47,717, whereas EZH2 overexpression increased the number of peaks to 72,083. The annotation of H3K27me3 peaks showed a broad distribution across intergenic, intronic, and promoter regions. The proportion of promoter-associated H3K27me3 peaks was 31.12% in control cells, 29.12% after EZH2 knockdown, and 32.15% after EZH2 overexpression (Figure 4A,B). Although these changes in the promoter-associated peak proportion were modest, they suggest that EZH2 perturbation is associated with changes in H3K27me3 peak abundance and genomic distribution in PKCi-mESCs.

To further assess whether EZH2 perturbation affects TSS-associated H3K27me3 signals, we generated TSS-centered heatmaps and metagene profiles across genes. H3K27me3 signals were enriched around TSS regions in all groups, with a lower signal intensity after EZH2 knockdown and a higher signal intensity after EZH2 overexpression (Supplementary Figure S8A). These data indicate that changes in H3K27me3 following EZH2 perturbation are not restricted to the *Nanog* locus, but also involve broader promoter-proximal regions.

We next examined the *Nanog* locus, given its established role in pluripotency regulation. In control PKCi-mESCs, H3K27me3 signals were detected at two proximal upstream regions of *Nanog*, approximately −2.2 kb to −1.9 kb and −1.8 kb to −1.5 kb relative to the transcription start site. EZH2 knockdown was associated with reduced H3K27me3 enrichment at these regions, whereas EZH2 overexpression was associated with an increased H3K27me3 signal at the same regions (Figure 4C). The quantification of H3K27me3 coverage at the *Nanog* promoter showed that the relative signal decreased from 1.00 to 0.74 after EZH2 knockdown and increased to 1.19 after EZH2 overexpression (Figure 4D). These changes were consistent with the observed increase in *Nanog* expression after EZH2 knockdown and decrease after EZH2 overexpression. Thus, the *Nanog* promoter represents an H3K27me3-marked, EZH2-sensitive locus in PKCi-mESCs.

To determine whether similar H3K27me3 changes also occur at other pluripotency-associated genes, we examined the *Pou5f1*/*Oct4* locus. H3K27me3 signals at the *Pou5f1*/*Oct4* genomic region were also altered after EZH2 knockdown or overexpression (Supplementary Figure S8B), indicating that chromatin changes following EZH2 perturbation are not limited to *Nanog*. However, the *Oct4* mRNA expression was not significantly changed in our EZH2 perturbation experiments, suggesting that changes in H3K27me3 at individual loci do not necessarily produce proportional transcriptional responses. Together, these CUT&Tag results support a model in which EZH2 perturbation is associated with altered H3K27me3 deposition across promoter-proximal regions and at selected pluripotency-associated loci, with *Nanog* representing one important EZH2-sensitive downstream target in PKCi-mESCs.

## 4. Discussion

The chromatin structure and post-translational histone modifications profoundly influence transcription and gene expression. As a chromatin-modifying complex, PRCs plays an important role in regulating gene expression during mammalian development [40,41]. PRCs contain catalytic cores that bind accessory proteins to form distinct repressive complexes (i.e., PRC1 and PRC2) [42]. Among these complexes, EZH2 (the core catalytic subunit of PRC2) mediates H3K27me3, which plays an essential role in gene silencing and chromatin state regulation [43]. In this study, we profiled the expression of PRC2 core components in PKCi-mESCs and demonstrated that EZH2 acts as a critical epigenetic regulator of pluripotency in this context. While EZH2 represses *Nanog* via H3K27me3 deposition at its promoter, EZH2 also governs a broader transcriptional network controlling pluripotency and lineage commitment, with *Nanog* serving as one important downstream target. We acknowledge that the observed pluripotency and differentiation phenotypes may result from H3K27me3 redistribution and the coordinated regulation of multiple loci, rather than solely from local changes at *Nanog*. This important finding reveals a potential epigenetic mechanism by which PKC inhibition influences the poised state of mESCs. Future rescue experiments, such as *Nanog* restoration under EZH2 overexpression or targeted epigenome editing at the *Nanog* promoter, would be required to define the causal hierarchy more rigorously. Building upon the established role of NANOG as a master regulator of pluripotency [34,35,36], our findings demonstrate that EZH2-mediated repression of *Nanog* substantially modulates the balance between self-renewal and lineage commitment under PKCi treatment, with *Nanog* acting as a key node in this regulatory network.

The analysis of PRC2 core components in PKCi-mESCs revealed a significant increase in the expression of the functional subunit EZH2 at both the mRNA and protein levels compared with 2iL-mESCs (Figure 1). EZH2 catalyzes H3K27me3 [44]; yet, the elevated EZH2 in PKCi-mESCs did not trigger a global increase in H3K27me3. Instead, PKCi induced locus-specific redistribution. Compared with 2i-mESCs, PKCi-mESCs showed a reduced H3K27me3 signal in the promoter and proximal TSS regions, while H3K27me3 was relatively increased in the gene-body and 5′UTR regions. For stem-cell-specific genes, the H3K27me3 signal was markedly reduced near the proximal TSS in PKCi-mESCs, whereas the H3K4me3 and H3K27ac signals were enhanced [45]. These observations confirm that PKCi-mESCs exhibit H3K27me3 redistribution rather than a uniform global hyper-trimethylation. We therefore propose that PKC inhibition sustains mESC self-renewal by upregulating EZH2, which functions as the dominant PRC2 catalytic subunit in this setting due to its stronger enzymatic activity [19]. Additionally, EED is required for the stabilization of both EZH2 and SUZ12 [21] and is essential for the silencing of pluripotency genes during ESC differentiation [46]. Although *Eed* mRNA levels were lower in PKCi-mESCs than in 2iL-mESCs, the protein levels did not show a significant difference (Figure 1). RBBP7 binds to histones H3 and H4 and is necessary for nucleosome association [47]; it also contributes to transcriptional activation [48]. Although *Rbbp7* mRNA expression was reduced in PKCi-mESCs, the corresponding protein levels remained unchanged (Figure 1). These discrepancies between transcription and translation for EED and RBBP7 may be attributable to translation regulation or a low RNA threshold requirement for protein synthesis.

To clarify whether the PKC-inhibition-mediated upregulation of PRC2 core components contributes to the regulation of pluripotency in PKCi-mESCs, we performed gene interference experiments that knocked down EZH2. The results showed that EZH2 knockdown diminished global H3K27me3 levels and upregulated NANOG expression, accompanied by more AP-positive undifferentiated colonies, increased *Klf4* expression, and suppressed germ-layer-associated genes (Figure 2). While Polycomb complexes canonically repress developmental genes in mESCs [14], the downregulation of germ-layer-associated genes upon EZH2 knockdown may stem from culture conditions, signaling pathways [7], or additional epigenetic mechanisms [49,50]. Conversely, EZH2 overexpression increased H3K27me3, reduced NANOG, decreased the proportion of undifferentiated colonies, downregulated *Sox2*, *Klf4*, *Fgf4*, and *Esrrb*, and broadly activated germ-layer-associated genes (Figure 3). Our findings appear to contrast with seminal studies demonstrating that EZH2 is essential for maintaining ESC identity and the poised state of developmental regulators [14,28]. In those studies, EZH2 knockout or knockdown in serum/LIF-cultured mESCs led to the loss of pluripotency and the derepression of differentiation genes. However, the PKCi culture system differs substantially from serum/LIF or even 2iL conditions. PKCi promotes a more naïve pluripotent state with unique epigenetic features, including an altered H3K27me3 distribution [45] and the upregulation of EZH2. Under PKCi, EZH2 knockdown does not cause differentiation; rather, it enhances self-renewal and suppresses lineage markers. These results indicate that EZH2 functions as a negative modulator of self-renewal in PKCi-mESCs. We propose that the PKCi environment rewires the pluripotency circuitry such that EZH2-mediated *Nanog* repression becomes limiting for self-renewal; lifting this repression via EZH2 knockdown reinforces pluripotency and overrides developmental gene derepression. Thus, the function of EZH2 may be highly context-dependent, and our results do not contradict the previous work but rather may extend it to a distinct pluripotency culture condition.

Previous studies have shown that EZH2, as a direct regulator of NANOG, coordinates NANOG expression and maintains the balance between self-renewal and differentiation in stem cells [36]. A critical aspect of our work is the investigation of this regulatory mode specifically within the PKCi system, which exhibits enhanced naïve characteristics compared to serum/LIF cultures [6]. CUT&Tag profiling confirmed that EZH2 directly governs H3K27me3 deposition at the *Nanog* promoter near the transcription start site (Figure 4). EZH2 knockdown reduces H3K27me3 enrichment at this locus, while overexpression increases it, in strict correlation with *Nanog* transcript and protein levels. Although H3K27me3 changes also occur at other pluripotency loci such as *Oct4*, the *Oct4* expression was not significantly altered, indicating the locus-specific sensitivity to EZH2-dependent repression. The *Nanog* promoter thus represents a key EZH2-sensitive epigenetic switch in PKCi-mESCs. In addition, other studies have shown that NANOG represses germ-layer-associated genes [33]. We observed that EZH2 knockdown reduced H3K27me3 levels, yet unexpectedly downregulated the expression of multiple germ-layer-associated genes, whereas EZH2 overexpression led to their marked upregulation. These observations appear to contradict the canonical model whereby the loss of H3K27me3 is associated with the derepression of developmental genes. One plausible explanation is that the elevated NANOG expression upon EZH2 knockdown (Figure 2D) mediates the repression of these differentiation-related genes [33], although direct evidence from NANOG chromatin binding assays is currently lacking. Alternative mechanisms may also contribute: (i) the moderate reduction in H3K27me3 (approximately 45%) may be insufficient to trigger a full derepression in the absence of lineage-specifying activators; (ii) other repressive epigenetic modifications, such as H3K9me3, may sustain gene silencing; and (iii) EZH2 knockdown may perturb additional transcriptional regulators beyond the PRC2-H3K27me3 axis. Collectively, these findings raise the possibility that the observed transcriptional changes could result from a combination of direct and indirect regulatory effects. Future studies incorporating NANOG ChIP-seq and functional rescue assays will be required to formally establish causality and clarify the underlying regulatory hierarchy.

NANOG also participates in interconnected regulatory circuits with other core pluripotency factors like OCT4 and SOX2, which collaboratively maintain the pluripotency network and suppress differentiation programs [33]. While we observed the specific downregulation of *Sox2* upon EZH2 overexpression (Figure 3F), changes in *Oct4* expression were not significant in our knockdown/overexpression models. This selectivity could arise from the differential susceptibility of these core factors to EZH2-mediated repression or the involvement of alternative regulatory mechanisms specific to the PKCi environment. Notably, the knockdown of EZH2 significantly upregulated the naïve-state-specific marker *Klf4* (Figure 2F), while the overexpression of EZH2 significantly suppressed naïve-state-specific markers including *Klf4*, *Fgf4*, and *Esrrb* (Figure 3F). This change likely arises from both NANOG-dependent network effects and direct PRC2-mediated repression. Alternatively, EZH2 might directly target the promoters of these naïve pluripotency genes via H3K27me3 modification, a possibility supported by the known role of PRC2 in repressing developmental regulators [15]. Collectively, these findings highlight the unique regulatory role of the PRC2 subunit EZH2 in balancing pluripotency and differentiation in PKCi-mESCs. EZH2-mediated H3K27me3 modification regulates the expression of core pluripotency genes (*Nanog*) and sustains self-renewal under PKCi conditions. In this study, we did not conduct an in-depth analysis of other pluripotency genes such as *Klf4*, *Esrrb*, and *Fgf4*, warranting a future investigation of the mechanism by which the regulatory networks of pluripotency factors maintain the self-renewal of PKCi-mESCs. The future genome-wide profiling of EZH2/H3K27me3 targets in PKCi-mESCs will elucidate the full scope of its regulatory influence. In addition, our data clearly establish EZH2 as a key epigenetic regulator balancing self-renewal and tri-lineage differentiation in PKCi-mESCs. However, these data are primarily supported by the qPCR expression profiling of germ layer markers and AP staining, rather than by direct functional differentiation assays. Future work will incorporate EB formation and teratoma assays to further validate the functional role of EZH2 in governing the tri-lineage differentiation potential.

## 5. Conclusions

Self-renewal and differentiation decisions in ESCs can be governed by epigenetic regulation, whereby signaling pathways directly or indirectly influence epigenetic modifiers and alter gene expression programs [51,52]. This study identifies a crucial epigenetic pathway regulating the pluripotent state in PKCi-mESCs. Specifically, PKC inhibition upregulates EZH2, which represses the core pluripotency gene *Nanog* via H3K27me3 deposition at its promoter. Beyond *Nanog*, EZH2 orchestrates a broader transcriptional network involving pluripotency and lineage-specific genes, indicating that *Nanog* serves as a critical downstream target through which EZH2 fine-tunes pluripotency and differentiation in PKCi-mESCs. Our data support a model in which EZH2 drives cell fate decisions via broad epigenetic reprogramming across multiple genomic targets, rather than through a simple linear cascade centered on a single gene. These findings reveal the unique regulatory dynamics of EZH2 within the PKCi system relative to the classical 2iL system, and provide mechanistic insight into the epigenetic control of self-renewal and pluripotency in PKCi-mESCs. While a direct functional assessment of the tri-lineage differentiation potential via EB or teratoma assays was not performed, our qPCR and AP staining data consistently indicate that EZH2 modulates pluripotency and differentiation gene programs in PKCi-mESCs.

## Figures and Tables

**Table 1:** qPCR primer sequences and sizes of amplified DNA products.

Gene	Forward Primer (5′–3′)	Reverse Primer (5′–3′)	Product Size (bp)
*Bmp4*	ATCACGAAGAACATCTGGAG	GAGATCACCTCATTCTCTGG	100
*cTnT*	AGACTGGAGTGAAGAAGAGGAGGAC	CTGGGCTTGGGTTTGGTGT	186
*Desmin*	AGAAAGTGCATGAAGAGGAG	CCTCAGAGATGTTCTTAGCC	156
*Eed*	AGCCACCCTCTATTAGCAGTT	GCCACAAGAGTGTCTGTTTGGA	206
*Esrrb*	AACAGCCCCTACCTGAACCT	CTCATCTGGTCCCCAAGTGT	245
*Ezh1*	TTCGTGTCCAGCCTGTTCA	AGTGTTCAGAGACCGCATCA	125
*Ezh2*	GGACCACAGTGTTACCAGCA	GGGCGTTTAGGTGGTGTCTT	167
*Fgf4*	GTGGTGAGCATCTTCGGAGTGG	GCGTAGGATTCGTAGGCGTTGT	146
*Fgf5*	GCTCGGAACATAGCAGTTTC	CCGTAAATTTGGCTTAACACAC	151
*Foxa2*	CACCTGAGTCCGAGTCTGAG	CGAGTTCATGTTGGCGTAGG	84
*Gata4*	TCTCACTATGGGCACAGCAG	GCGATGTCTGAGTGACAGGA	136
*Gata6*	CGGTCTCTACAGCAAGATGAAT	TGGTTGTGGTGTGACAGTTG	113
*Klf4*	ACTGTCACCCTGGCCTGCCTCT	CCCTCTTTGGCTTGGGCTCCT	165
*Nanog*	GTCTGATTCAGGGCTCAGCA	AAGGCTTCCAGATGCGTTCA	92
*Nestin*	CTCGAGCAGGAAGTGGTAGG	TTGGGACCAGGGACTGTTAG	353
*Oct4*	GAAGCAGAAGAGGATCACCTTG	TTCTTAAGGCTGAGCTGCAAG	129
*Pax6*	CGGAAGCTGCAAAGAAATAG	CCTGTATTCTTGCTTCAGGT	145
*Rex1*	GCCAGTCCAGAATACCAGAGT	AGCCATCTTCCTCAGTCTCG	92
*Rbbp4*	GTGCTTCAGATGACCATACCAT	CGTCCTCCACTACTGCTGTA	114
*Rbbp7*	CTGTGGAGGAGCGTGTCAT	CCAGCACTAGCCAATGAAGG	174
*Sox1*	GGCCGAGTGGAAGGTCATGT	TCCGGGTGTTCCTTCATGTG	93
*Sox2*	ACAGCATGTCCTACTCGCAG	ATGCTGATCATGTCCCGGAG	160
*Sox17*	GCGTGGAGCAGGACCCGGCTTTCTT	GGACACTGCATAGTCCGAGACTGGA	101
*Suz12*	CCACAGCAGGTTCATCTTCAA	GCATAGGAGCCATCATAACACT	90
*T*	ACCTATGCGGACAATTCATC	CAGACCAGAGACTGGGATAC	155
*β-Actin*	TGTTACCAACTGGGACGACA	GGGGTGTTGAAGGTCTCAAA	165

**Table 2:** Antibodies used for Western blot analyses.

Antibodies	Source	Identifier
Rabbit polyclonal anti-EED	ABclonal Technology	Catalog NO.: A12773
Rabbit polyclonal anti-EZH1	ABclonal Technology	Catalog NO.: A5818
Rabbit polyclonal anti-EZH2	ABclonal Technology	Catalog NO.: A5743
Rabbit polyclonal anti-H3K27me3	ABclonal Technology	Catalog NO.: A16199
Rabbit monoclonal anti-NANOG	Abcam Technology	Catalog NO.: ab214549
Rabbit polyclonal anti-RBBP7	ABclonal Technology	Catalog NO.: A13456
Rabbit monoclonal anti-β-Actin	ABclonal Technology	Catalog NO.: AC026

## Data Availability

All data related to this paper may be requested from the corresponding author upon reasonable request.

## Supplementary Materials

The following supporting information can be downloaded at: https://www.mdpi.com/article/10.3390/biology15110880/s1, Figures S1–S8: WB, CUT&Tag.
