# Supplementary material for: EZH2 Regulates the Pluripotency of Mouse Embryonic Stem Cells by Modulating Nanog Expression Under PKC Inhibition
Source: Biology (Basel). 2026 Jun 2;15(11):880. doi: 10.3390/biology15110880 (PMC13255847; doi:10.3390/biology15110880)
Supplement: Supplementary file 1 [file biology-15-00880-s001.zip › biology-4284403-supplementary.pdf]

EZH2 Regulates the Pluripotency of Mouse Embryonic Stem Cells by Modulating *Nanog* Expression Under PKC Inhibition

Fangfang Wu [1, a], Zhihui Liu [2, a], Yuan Gao [3], Jinshan Li [3], Xiao Chen [3], Xiyue Wang [4], Lanjun Liu [5], *, Fuliang Du [3], *

[1]School of Nursing and Health, Shanghai Zhongqiao Vocational and Technical University, Shanghai 200540, China
[2]College of Animal Science and Technology, Hebei Agricultural University, Baoding, 071000, Hebei, PR China
[3]Jiangsu Key Laboratory for Molecular and Medical Biotechnology, College of Life Sciences, Nanjing Normal University, Nanjing 210046, China
[4]Children's Research Institute, University of Texas Southwestern Medical Center, Dallas, TX 75390, USA
[5]Chengdu Institute of Biological Products Co., Ltd., 379, 3rd Section, Jinhua Road, Jinjiang District, Chengdu, 610023, China
[a]These authors contributed equally: Fangfang Wu, Zhihui Liu.
*Correspondence: author: 1. Fuliang Du, PhD, Professor Email: fuliangd@njnu.edu.cn 2. Lanjun Liu, Director E-mail: liulanjun@sinopharm.com

Figure S1

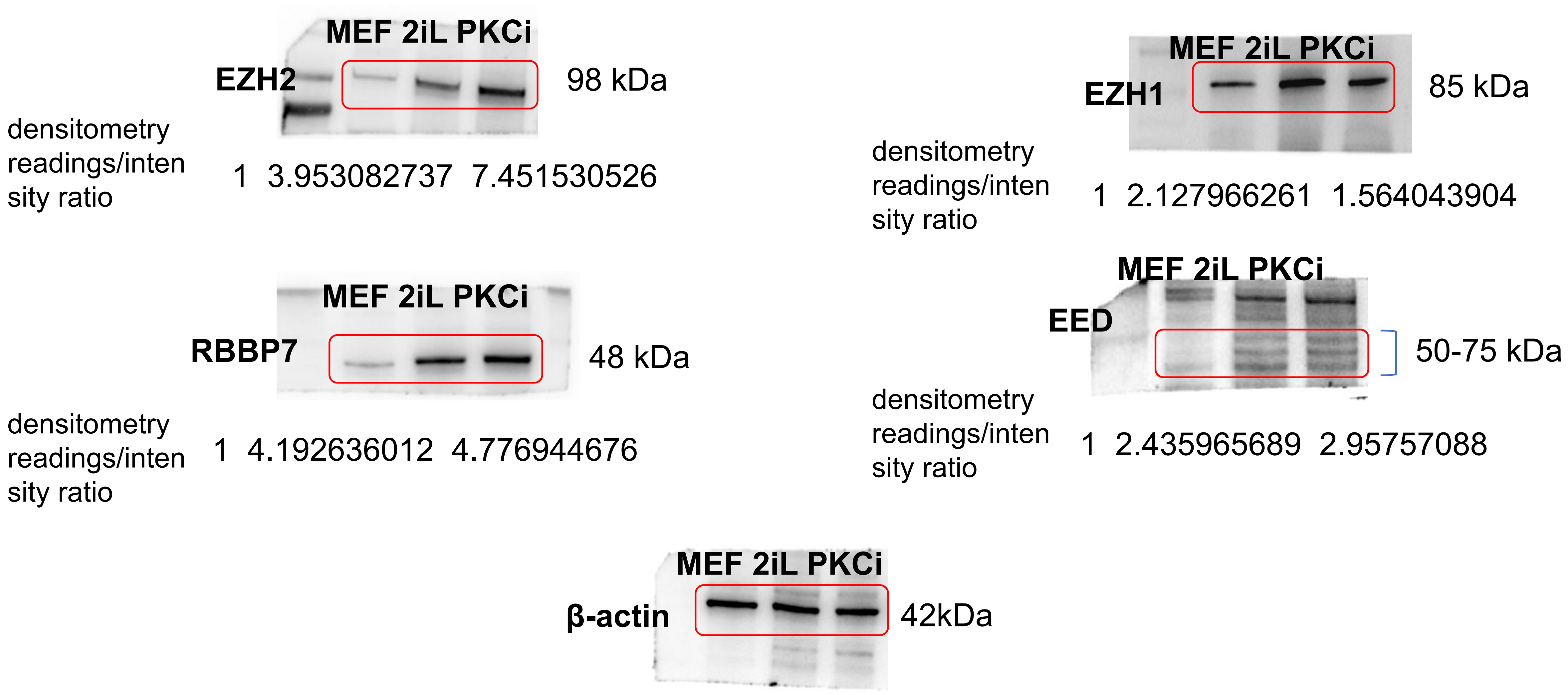
MEF 2iL PKCi
EZH2
98 kDa
densitometry readings/intensity ratio
1 3.953082737 7.451530526
MEF 2iL PKCi
EZH1
85 kDa
densitometry readings/intensity ratio
1 2.127966261 1.564043904
MEF 2iL PKCi
RBBP7
48 kDa
densitometry readings/intensity ratio
1 4.192636012 4.776944676
MEF 2iL PKCi
EED
50-75 kDa
densitometry readings/intensity ratio
1 2.435965689 2.95757088
MEF 2iL PKCi
β-actin
42kDa

Figure S2

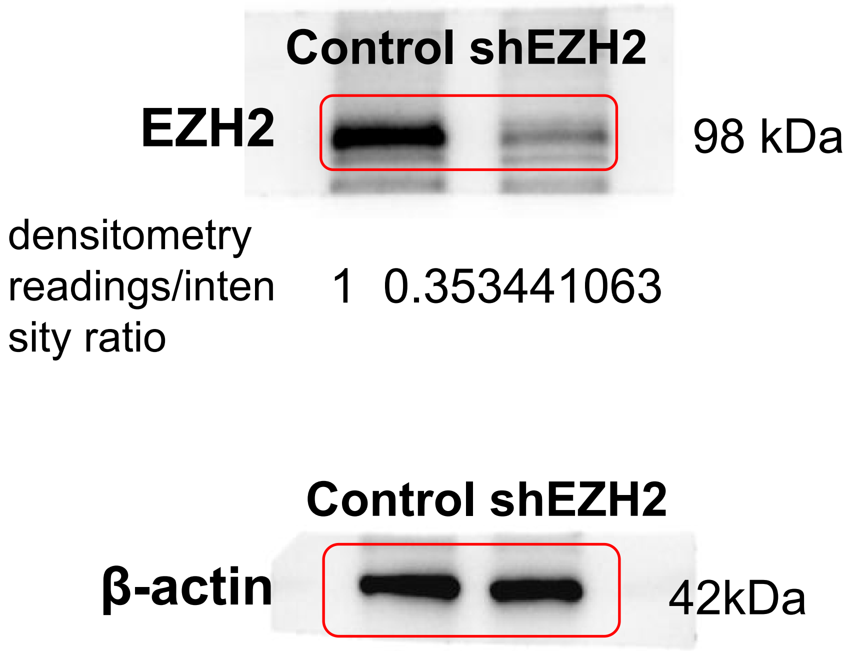
Control shEZH2
EZH2
98 kDa
densitometry readings/intensity ratio
1 0.353441063
Control shEZH2
β-actin
42kDa

Figure S3

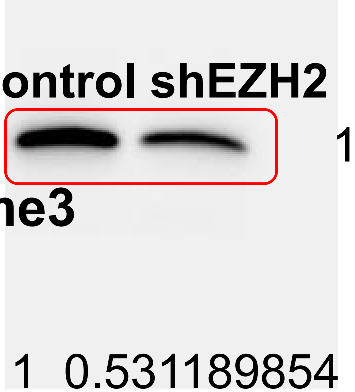

densitometry readings/intensity ratio 1 0.531189854

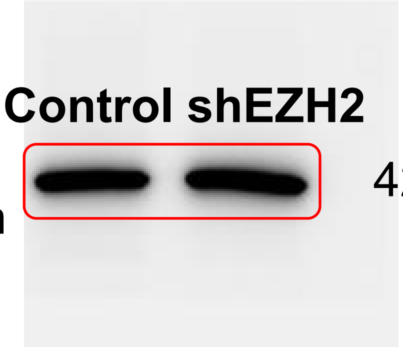

Figure S4

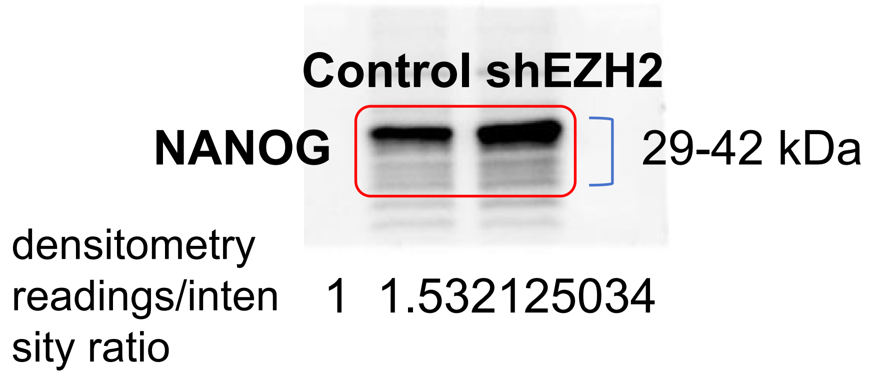
Control shEZH2
NANOG
29-42 kDa
densitometry readings/intensity ratio
1 1.532125034

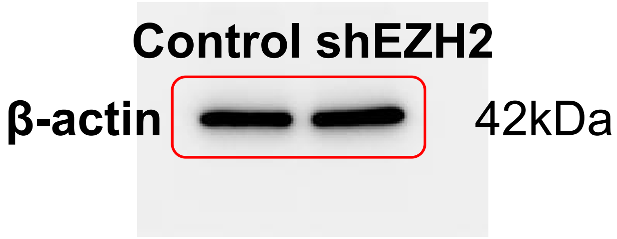
Control shEZH2
β-actin
42kDa

Figure S5

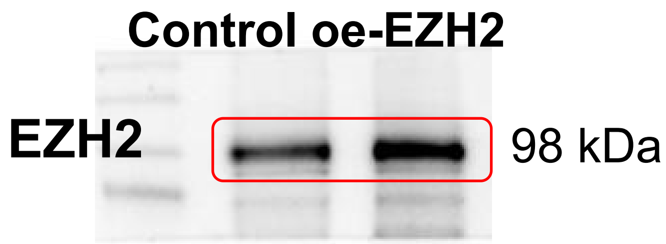

densitometry readings/intensity ratio 1 1.558882014

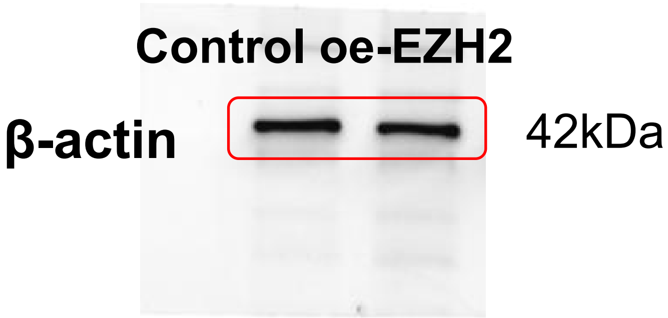

Figure S6

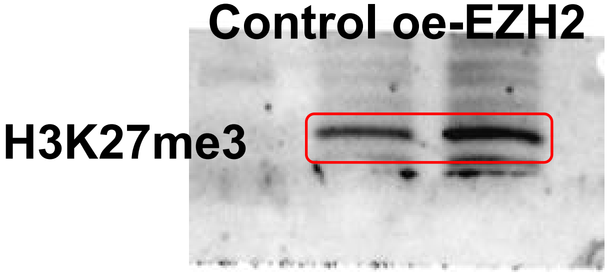

densitometry readings/intensity ratio 1 1.526967169

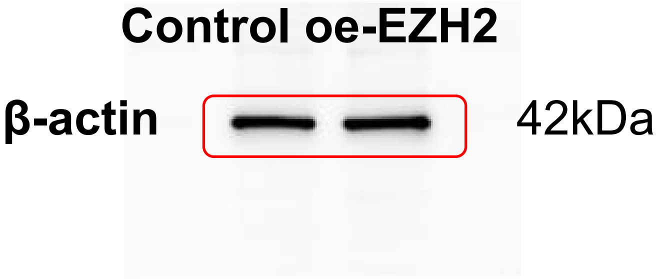

Figure S7

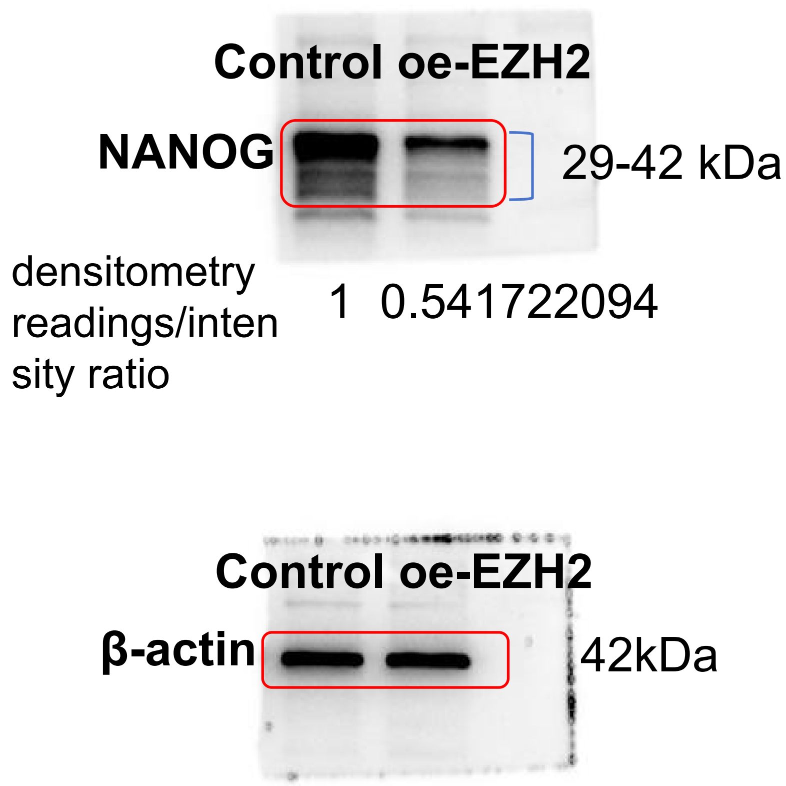
Control oe-EZH2
NANOG
29-42 kDa
densitometry readings/intensity ratio
1 0.541722094
Control oe-EZH2
β-actin
42kDa

Figure S8

A

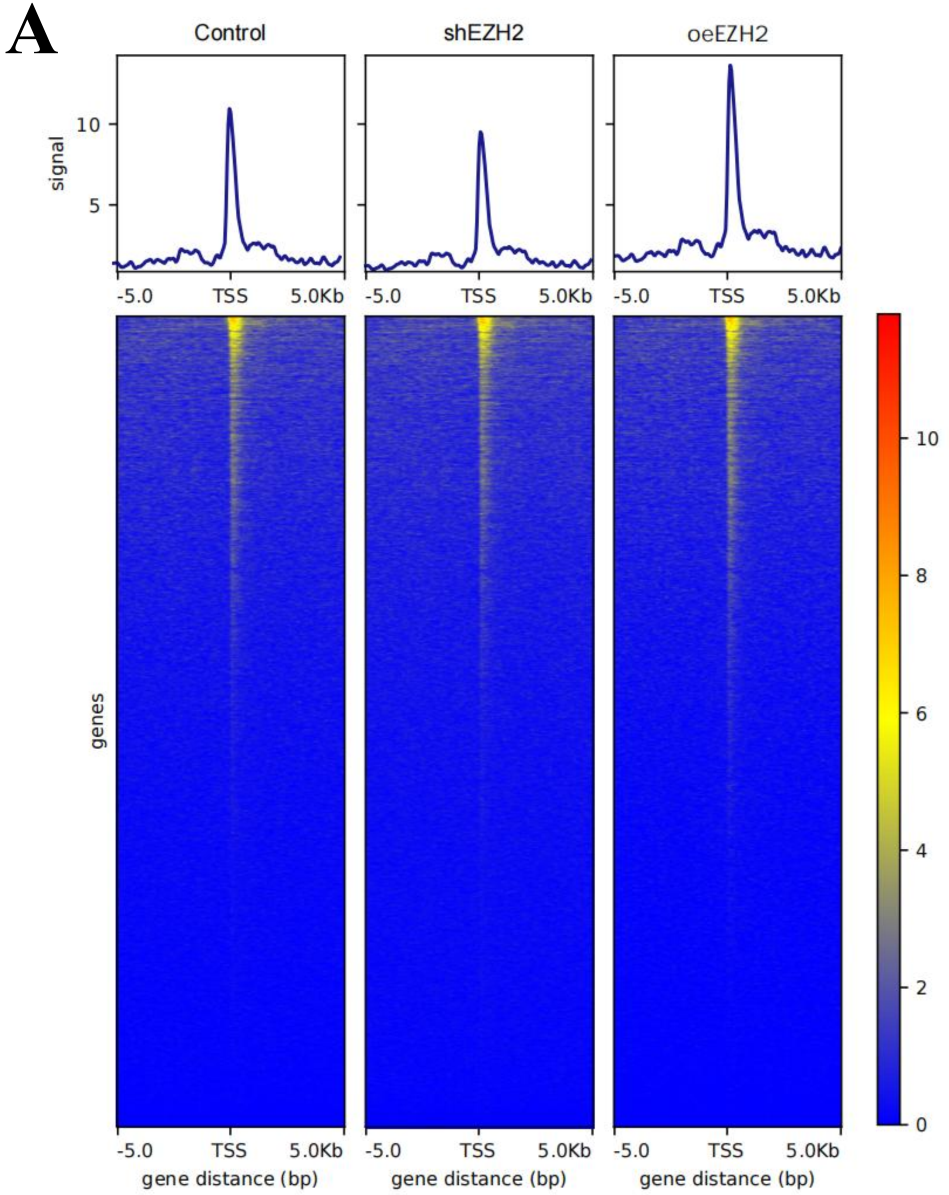

B

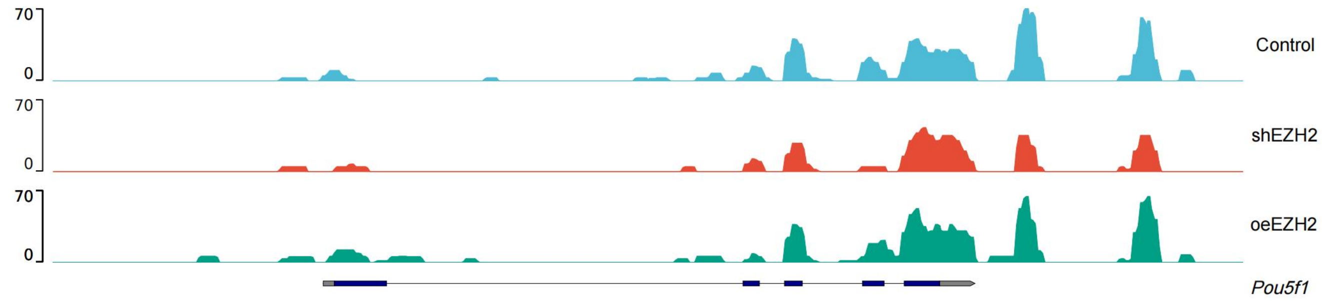

Supplementary Figure S8. TSS-centered H3K27me3 CUT&Tag profiles and representative pluripotency-gene loci in PKCi-mESCs after EZH2 perturbation.

(A) TSS-centered heatmaps and metagene profiles of H3K27me3 signals in control, shEZH2, and oe-EZH2 PKCi-mESCs.

(B) Genome browser view showing H3K27me3 signals at the *Pou5f1*/*Oct4* locus in control, shEZH2, and oe-EZH2 PKCi-mESCs.
